# Diagnostic Accuracy of Cancer Antigen 15–3 as a Seromarker Among Recurrent Breast Carcinoma in Bangladesh

**DOI:** 10.7759/cureus.68448

**Published:** 2024-09-02

**Authors:** Rawnok Jahan Kabir, Refoyez Mahmud, Md Enamul Kabir, Abdullah Md Abu Ayub Ansary, Salma Sultana, Mayisha Rahman, Dipannita Adhikary, Adneen Moureen, Redoy Ranjan, Md Abdullah Yusuf

**Affiliations:** 1 Surgery, Siraj-Khaleda Memorial Cantonment Board General Hospital, Dhaka, BGD; 2 Pediatric Surgery, Bangladesh Shishu Hospital and Institute, Dhaka, BGD; 3 Surgery, Marks Medical College and Hospital, Dhaka, BGD; 4 Surgery, Bangabandhu Sheikh Mujib Medical University, Dhaka, BGD; 5 Surgery, Dhaka Medical College and Hospital, Dhaka, BGD; 6 Biochemistry and Molecular Biology, Georgetown University, Dhaka, BGD; 7 Biological Sciences, Royal Holloway University College London, London, GBR; 8 Tuberculosis (TB) New Technologies and Diagnostics (Bangladesh), United States Agency for International Development (USAID), Dhaka, BGD; 9 Cardiac Surgery, St. Georges University Hospital NHS Foundation Trust, London, GBR; 10 Cardiac Surgery, Bangabandhu Sheikh Mujib Medical University, Dhaka, BGD; 11 Biological Science, Royal Holloway University of London, London, GBR; 12 Microbiology, National Institute of Neurosciences and Hospital, Dhaka, BGD

**Keywords:** high risk breast cancer, breast cancer research, recurrence predictors, ca breast prognostic markers, ca 15-3

## Abstract

Background: The diagnosis of recurrent breast carcinoma is crucial for patient treatment. The present study aimed to assess the diagnostic accuracy of cancer antigen 15-3 (CA 15-3) as a sero-marker among recurrent breast carcinoma patients.

Methods: This prospective observational study evaluated the serum CA 15-3 among women (age ≥18 years) with recurrent breast carcinoma. The CA 15-3 was measured by the enzyme-linked immunosorbent assay (ELISA), and concentrations were stratified using a cut-off value of 30 U/mL. The receiver operating characteristic (ROC) curve observed that the sensitivity and specificity of the CA 15-3 cut-off value and the area under the AUROC curve demonstrate the goodness-of-fit of the prediction model.

Results: A total of 50 patients were recruited, with a mean age of 48.4 ±9.7years. The majority (n=28, 56.0%) of patients were 41 to 50 years old. Further, a total of 42 (84%) patients had high serum levels of CA 15-3, with a mean value of 72.7±9.5 U/mL. At the cut-off level of 30 U/mL, the ROC curve demonstrated sensitivity, specificity, positive predictive value, and negative predictive value of 95.7%, 69.4%, 84.1%, and 72.8%, respectively, to diagnose recurrent breast carcinoma. Nonetheless, the area under the ROC (AUROC) curve was 0.712, indicating a satisfactory fit for the prediction model.

Conclusion: We found that CA 15-3 level ≥30 U/mL is highly sensitive and specific as a seromarker for detecting recurrent breast cancer among the Bangladeshi population. We recommend routinely monitoring breast cancer survivors using CA 15-3 biomarkers.

## Introduction

Serum cancer antigen 15-3 (CA 15-3) glycoprotein secreted by breast cancer cells is an established prognostic biomarker in breast cancer patients [1,2]. Studies have shown that CA 15-3 levels may provide helpful information for diagnosing and predicting recurrent breast cancer [2,3]. Previous analysis of recurrent breast cancer shows that elevated CA 15-3 level is predictive of a poor response to chemotherapy, and the diagnosis of recurrence is confirmed in about 37.0% of cases [3-5]. Nonetheless, a recent study on recurrent breast cancer following radical mastectomy found that elevated levels of CA 15-3 may predict relapse and shorter progression-free survival [6].

Recurrent breast cancer is a significant healthcare burden in women, and its incidence has been increasing. CA 15-3 has been a widely used sero-marker in the clinical fields to monitor treatment prognosis, recurrence, and treatment failure of breast cancer [6-8]. In a study, Kurebayashi et al. [7] demonstrated that CA 15-3 has a potential role in monitoring treatment response to chemotherapy in breast cancer patients, reinforced by Duffy et al. [8] among advanced and recurrent breast cancer patients. Early diagnosis of recurrent breast cancer using a cost-effective tumour marker can prevent further complications and mortality, making it a routine recommendation for patients [9-11].

This study observed the diagnostic accuracy of CA 15-3 at 30 U/mL as a sero-marker among recurrent breast carcinoma patients in Bangladesh.

## Materials and methods

Study population

This prospective observational cohort study recruited women (age ≥18 years) with recurrent breast carcinoma following adjuvant therapy among the Bangladeshi population. This study recruited participants between 2021 and 2022 at the Department of Surgery and Radiotherapy, Dhaka Medical College Hospital, Bangladesh, and the Department of Surgical Oncology at the National Institute of Cancer Research and Hospital, Dhaka, Bangladesh. This study applied a purposive sampling technique, and the histological diagnosis was established either through a core or excisional biopsy. Women aged 18 years or older with recurrent breast carcinoma after completion of adjuvant or neoadjuvant therapy were included in the study. However, patients who had not yet completed adjuvant therapy and males were excluded from this study.

During follow-up, serum CA 15-3 was measured by the enzyme-linked immunosorbent assay (ELISA) method, and the mean follow-up period was 41.4 ± 15.3 months. We extrapolated CA 15-3 concentrations and stratified them using a cut-off value of 30 U/mL as previously described [4]. Ethical clearance was obtained from the institutional review board of Dhaka Medical College and Hospital, Dhaka, Bangladesh (ERC-DMC/ECC/2019/366), and informed consent was obtained from each study subject. Data were collected and recorded in structured case report form and encrypted.

Statistical analysis

The data were processed and analysed using computer software SPSS v28.0 (Statistical Package for Social Sciences), and the results were presented in tables and figures as appropriate. Students’ t-tests did statistical analysis for quantitative variables and the Chi-square test for qualitative variables. Furthermore, we utilised a receiver operating characteristic (ROC) curve to observe the accuracy of the prediction model, the negative predicted value (NPV) and positive predicted value (PPV), as well as the sensitivity and specificity of the aforementioned cut-off value. The area under the ROC curve demonstrates the goodness-of-fit of the prediction model. A probability value <0.05 was considered as a level of statistical significance, and a 95% confidence interval was noted.

## Results

A total of 50 patients were recruited for this study after fulfilling the inclusion and exclusion criteria, with a mean age of 48.4 ± 9.7 years. A total of 28 (56.0%) patients were 41 to 50 years old, followed by 12 (24.0%) patients aged 51 to 60. The cut-off point of the CA 15-3 level was considered 30 U/mL, and we found 42 (82%) patients had high serum levels of CA 15-3 with a mean value of 72.7 ± 9.5 U/mL (Table 1).

**Table 1:** Distribution of age and CA15-3 level among the study subjects (n=50)

Variables		Frequency	Mean ± SD
Age	18–30 years	0 (0%)	48.4±9.7
	31–40 years	10 (20%)	
	41–50 years	28 (56%)	
	51–60 years	12 (24%)	
CA15-3 Level	<30 U/mL	8 (16%)	9.3±2.2
	≥30 U/mL	42 (84%)	72.7±9.5

Furthermore, the sensitivity, specificity, positive predictive value, and negative predictive values at the cut-off level of 30 U/mL or more were 95.7%, 69.4%, 84.1%, and 72.8%, respectively, to predict the recurrence of breast cancer (Table 2).

**Table 2:** Validity parameters of the prognostic value of CA 15-3 levels in predicting recurrent breast cancer NPV: negative predictive value; PPV: positive predictive value

Cut-off value	Sensitivity	Specificity	PPV	NPV	Accuracy
20	98.5%	68.9%	90.8%	63.6%	94.71%
30	95.7%	69.4%	84.1%	72.8%	90.50%
40	86.5%	78.1%	83.6%	58.4%	77.82%

**Figure 1:**
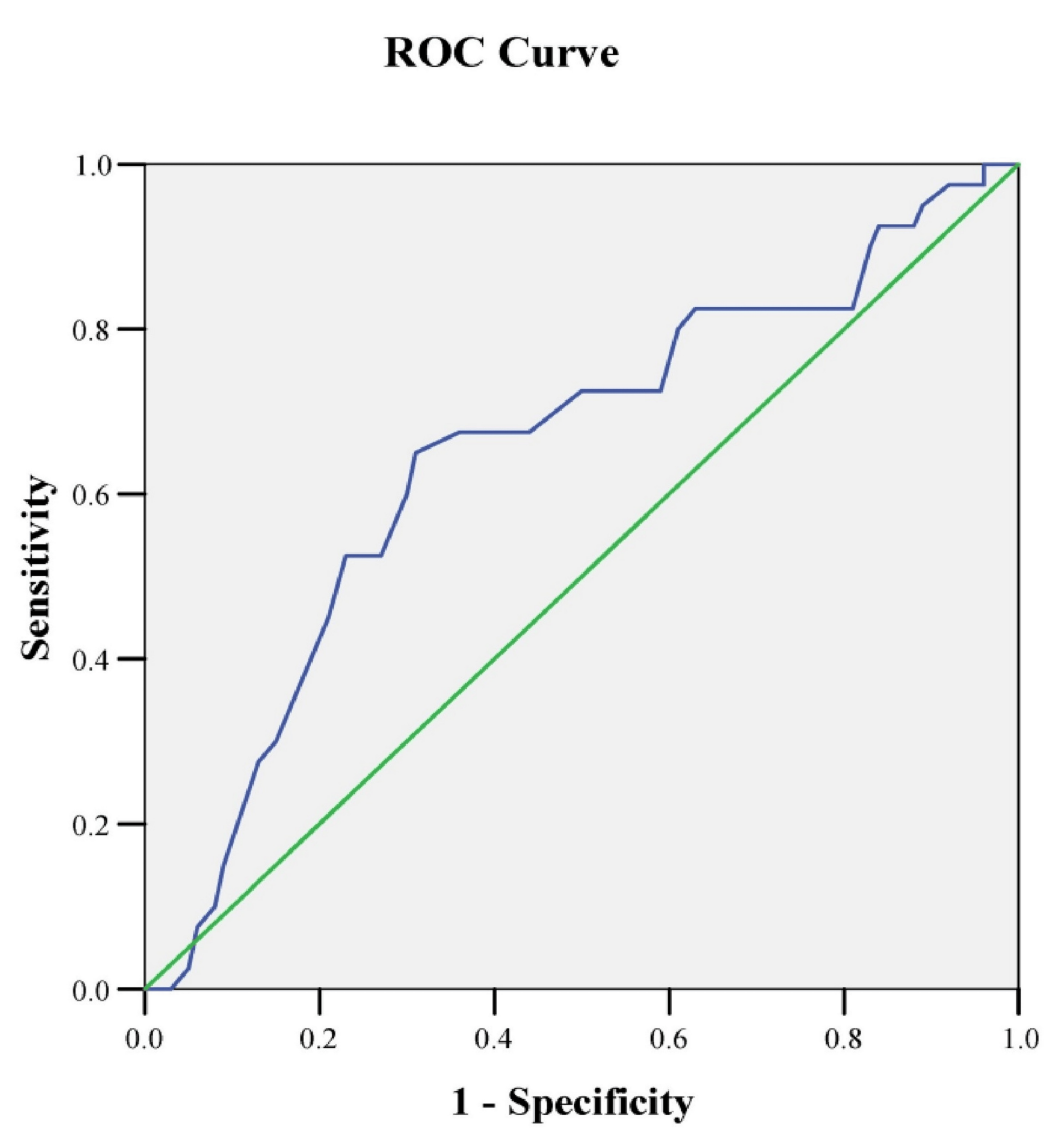
The receiver operating characteristic (ROC) curve of CA 15-3 predicting recurrent breast cancer

The prediction model's goodness-of-fit, represented by an area under the receiver operating characteristic (AUROC) curve, was 0.712, with sensitivity and specificity of 68.5% and 38.2%, respectively (Figure 1).

## Discussion

This study observed that high CA 15-3 levels predict higher recurrence rates and poor response to adjuvant therapy, helping in early detection of recurrence and improving treatment prognosis. Despite technological advancements in early intervention, many breast cancer patients still fear recurrent disease [3]. Breast cancer recurrence presents a significant clinical challenge that is not well understood, and researchers have studied multiple factors to predict the likelihood of disease recurrence [12]. Currently, approximately 40% of breast cancer patients experience a recurrence, leading to a high mortality rate, making it the leading cause of cancer-related death in women [13-15]. The occurrence and location of recurrences depend on the initial tumour stage, previous treatment, tumour biology, and the accuracy of the diagnosis [16]. Although the main prognostic factor in breast cancer is the status of axillary nodes, serum tumour marker levels are also potentially applicable [13]. Tumour markers CA 15-3 are currently used in detecting recurrence and monitoring treatment response to therapy, especially in patients with advanced disease, supporting study findings [16-18].

Recurrent breast cancer with high CA 15-3 levels predicts a poor response to chemotherapy and reduced disease-free survival in locally advanced breast cancer (LABC), along with lymphovascular invasion and human epidermal growth factor receptor (HER) 2 status [10]. A previous study [11] found that using CA 15-3 for follow-up in breast cancer patients increased the recurrence diagnosis rate by 37%, supported by Duffy, who concluded that CA 15-3 is most critical for monitoring therapy in advanced breast cancer patients [19]. However, similar to our study findings, previous studies have also found an association between elevated CA 15-3 levels and predicting a poor response to primary treatment or recurrence in locally advanced breast cancer [7,8,10]. Further, Kurebayashi et al. found that a reduction of over 20.0% in CA 15-3 levels post-treatment predicted a favourable outcome for disease progression during systemic therapy [7].

Although the American Society of Clinical Oncology (ASCO) does not recommend the routine use of CA 15-3 in LABC, the National Academy of Clinical Biochemistry (NACB) and the European Group on Tumour Markers (EGTM) recommend using CA 15-3 to monitor therapy in LABC [17,20]. Nevertheless, De La Lande et al. found a link between CA 15-3 lead time and prognosis, with high CA 15-3 levels predicting recurrence after primary treatment [21]. Additionally, several existing studies observed that elevated CA15-3 levels after mastectomy and adjuvant therapy were independent predictors of poor outcomes [6,8,10].

Despite the diagnostic accuracy of CA 15-3 in the current study, we need to acknowledge the limitations of the small sample size and observational nature of the study. The study's small sample size (n=50) limits the generalisability of the findings, making it difficult to apply the results to a broader population. The absence of a control group, such as patients without recurrent breast cancer, weakens the ability to fully assess the diagnostic accuracy of CA 15-3. However, considering the rare occurrence of breast cancer, the potential for outcome bias is mitigated. Despite purposive sampling being prone to bias as participant selection depends on investigator judgement and is not randomly assigned, it is effective, and potential bias is mitigated because of a small sample due to the rare recurrence of breast cancer. Since the study was conducted solely in Bangladesh, the findings may not be applicable to other populations with different demographic and genetic profiles. Additionally, the study does not account for potential confounding factors, such as variations in treatment regimens, that could impact CA 15-3 levels and the recurrence of breast cancer. Finally, the lack of detailed information on primary breast cancer characteristics, such as tumour size and lymph node status, hinders the ability to correlate disease severity with CA 15-3 levels.

## Conclusions

We found that approximately 85% of women with recurrent breast cancer have CA 15-3 levels of ≥30 U/mL, showing high sensitivity but low specificity in detecting recurrent breast cancer. We recommend using CA 15-3 biomarkers routinely during follow-up to monitor the recurrence among breast cancer survivors. Additionally, a large-scale randomised control trial or observational cohort study is needed to evaluate CA 15-3 levels in recurrent breast cancer to validate our findings.
